# Acting upon threat: Electrocortical, autonomic, and oculomotor responses in anticipation of avoidable and inevitable threats

**DOI:** 10.1162/IMAG.a.1318

**Published:** 2026-07-29

**Authors:** Janna Teigeler, Yannik Stegmann, Matthias Gamer

**Affiliations:** Experimental Clinical Psychology, Department of Psychology, University of Würzburg, Würzburg, Germany

**Keywords:** alpha, threat, eye tracking, attentive immobility, autonomic, EEG, oscillations

## Abstract

Attentive immobility is a defensive state that prepares organisms for adaptive responses to imminent threats. In humans, it has been associated with a multimodal pattern of heart rate bradycardia, elevated skin conductance, freezing of gaze, and reductions in visuocortical alpha power to enhance perceptual processing and action preparation. However, the extent to which these processes are shaped by threat intensity or rather reflect stereotypic response patterns remains elusive. Here we combined avoidable and inevitable threats with different shock intensities to dissociate threat intensity from action preparation in defensive responding. Forty-eight participants observed naturalistic images while electrocortical activity, heart rate, skin conductance, pupil dilation, and eye movements were recorded. The color and shape of the cue preceding each trial were indicative of the trial type and predicted an inevitable or avoidable high-intensity (painful) or low-intensity stimulation after the picture offset. Results showed stronger gaze centralization in avoidable trials, indicating enhanced action preparation. Skin conductance primarily reflected threat intensity, while heart rate, alpha power reduction, fixation durations, and pupil dilation varied with both threat intensity and action readiness. The interplay between these components suggests a need to move away from the notion of separate systems governing physiological and motor responses under threat. We argue for an integrative framework where defensive responses and motor preparation are driven by motivational relevance, which escalates with perceived imminence, harm potential, and escape possibility. These findings underscore the interconnected nature of affective and motor processes, offering new perspectives on human defensive behavior.

## 1 Introduction

The ability to exhibit a wide range of defensive behaviors in response to imminent danger is crucial for survival. When faced with threat, organisms adjust their responses based on the level of threat imminence, ranging from more reflexive and short-sighted actions for proximal threats to more deliberate actions that allow enhanced information processing and planning when the threat is more distal (Mobbs et al., 2020). For instance, when faced with imminent, inevitable danger, organisms might resort to fight or flight behaviors or engage in *tonic immobility*, a late-stage defensive state characterized by profound motor inhibition and reduced behavioral flexibility, as a last defensive resort (Fanselow, 1994; Mobbs et al., 2020; Volchan et al., 2017). By contrast, when a potential threat has been detected but the situation remains ambiguous and the danger might still be avoided with appropriate responses, organisms frequently enter a state of *attentive immobility* (Mobbs et al., 2020; Volchan et al., 2017). This state is often equated with restricted mobility, so-called freezing, especially in rodents (Hagenaars, Oitzl, et al., 2014). Because the term “freezing” has been used inconsistently to describe different immobility responses across threat contexts, we focus here on attentive immobility to clearly specify the defensive state of interest (Hagenaars, Oitzl, et al., 2014; Volchan et al., 2017). Attentive immobility describes a complex response pattern that is not limited to the motor component but is accompanied by specific autonomic changes, including fear bradycardia, and increased sympathetic arousal (Tovote et al., 2005). Importantly, unlike *tonic immobility*, attentive immobility represents an active, preparatory state that supports threat monitoring and action preparation, rather than a passive shutdown response occurring when defensive options are exhausted (Roelofs, 2017; Roelofs & Dayan, 2022).

Recent research has increasingly focused on attentive immobility in humans, as aberrant patterns have been implicated in various clinical conditions, such as PTSD (Fragkaki et al., 2017) or panic disorder (Lopes et al., 2009), as well as in subjects with a higher number of aversive life events (Hagenaars et al., 2012) and elevated levels of trait anxiety (Hashemi et al., 2021; Wada et al., 2001). Additionally, similarities in response patterns across species, including rodents, non-human, and human primates, have been observed: In humans, similar to other species, attentive immobility is characterized by motor inhibition and concurrent sympathetic and parasympathetic upregulation (Roelofs & Dayan, 2022). Even though motor inhibition is much more pronounced in rodents, studies using movement tracking found decreased body sway in response to uncertain threats (Hagenaars, Roelofs, et al., 2014; Roelofs et al., 2010), and motor inhibition further seems to extend to eye movements (“freezing of gaze”), as indicated by fewer and longer fixations as well as a more centralized visual exploration patterns (Merscher & Gamer, 2024a, 2024b; Merscher et al., 2022; Rösler & Gamer, 2019). Empirical evidence further suggests that attentive immobility in humans is associated with fear bradycardia and increased skin conductance levels (Gladwin et al., 2016; Löw et al., 2015; Merscher et al., 2022; Rösler & Gamer, 2019; Wendt et al., 2017), as well as enhanced perceptual sensitivity (Lojowska et al., 2015), and facilitated action preparation (Gladwin et al., 2016; Rösler & Gamer, 2019). A recent study investigating electrocortical activity during states of attentive immobility found pronounced suppression of visual alpha power (Stegmann et al., 2024), indicating heightened cortical arousal (Foxe & Snyder, 2011; Klimesch et al., 2007) and enhanced attentional processes (Kelly et al., 2006; Klimesch, 2012). Faster responses to escape from threat were predicted by a more centralized gaze, stronger heart rate deceleration, and increased suppression of alpha activity, highlighting the functional significance of these components (Stegmann et al., 2024). In summary, attentive immobility in humans has been associated with a complex multimodal pattern on the electrocortical, autonomic, and behavioral level, which is assumed to enhance perceptual processing and action preparation. These findings underscore the conceptualization of attentive immobility as an active, adaptive mechanism to cope with imminent threat (Roelofs, 2017).

A persistent challenge in understanding defensive responding lies in the fact that autonomic reactivity is not consistently threat specific. Measures such as heart rate deceleration, skin conductance level, pupillary responses, and body sway do not consistently distinguish between threatening, safe, or even rewarding contexts. In several task settings, these responses have instead been linked to the opportunity for action—such as quickly pressing a button or using a virtual gun to counter a potential opponent—suggesting that autonomic activity may reflect action readiness alongside, or independent of, threat processing (Gladwin et al., 2016; Löw et al., 2015; Merscher et al., 2022). On the one hand, these findings may suggest an intrinsic link between action preparation and threat-related processes (Stegmann et al., 2024). On the other hand, they might also reflect specific features of experimental paradigms focused on contrasting active and passive threat conditions (e.g., Rösler & Gamer, 2019; Stegmann et al., 2024). While comparisons between high- and low-threat conditions within active paradigms have shown that heart rate dynamics are potentiated by threat of shock (Gladwin et al., 2016; Hashemi et al., 2021), it remains unclear whether and how autonomic and neural markers are differentially affected by threat intensity and action possibility when these factors are manipulated orthogonally.

To address this research gap, we adapted an established paradigm (Rösler & Gamer, 2019; Stegmann et al., 2024), in which a color cue signals whether a naturalistic image is followed by an inevitable or avoidable shock, by introducing varying shock intensities to manipulate threat perception. We measured electrocortical, autonomic, and oculomotor responses in anticipation of avoidable and inevitable high-intensity (painful) or low-intensity electric stimuli. Overall, we expected that attentive immobility varies depending on both threat intensity and response availability of the situation. Based on prior work, we further expected that components previously linked to attentive immobility—particularly heart rate deceleration, gaze centralization, and alpha power suppression—would predict faster responses to escape from threat (Stegmann et al., 2024). A key open question was whether the strength and specificity of these associations depend on perceived threat intensity, or whether they primarily reflect response availability independent of intensity. Accordingly, a central aim of our study was to discern which aspects of the multimodal defensive response pattern are specific to perceived threat intensity, motor response preparation, or are influenced by both factors.

## 2 Methods

### 2.1 Transparency and openness

This study’s design, all hypotheses, and analyses were preregistered in December 2023 at https://osf.io/2kp4s. Aggregated datasets and the code for pre-processing of the data and all statistical analyses are available on the Open Science Framework at https://osf.io/f9hjx/.

### 2.2 Participants

We aimed at a sample size of *n* = 50 based on the power analysis of our previous experiments (Rösler & Gamer, 2019; Stegmann et al., 2024) and adjusted for the additional requirements of EEG studies. This sample size enables the detection of medium effect sizes in repeated measures analyses of variance (ANOVAs, *f* = 0.25) at an alpha level of 0.05, with a statistical power exceeding 0.95, assuming a correlation of *r* = 0.50 between factor levels. We collected data from 52 participants to account for expected data loss. In the end, four participants were excluded from all analyses due to technical issues (*n* = 1 subject underwent the first block two times instead of blocks 1 and 2), or not following instructions (*n* = 2 subjects consistently responded too early, before the picture offset; *n* = 1 subject responded during passive (inevitable) trials, resulting in empty cells in the inevitable threat condition). This led to a final sample size of *N* = 48 (26.8 ± 5.35 years old, 32 female, 15 male, 1 diverse), which still ensures a detection of medium effect sizes with a power greater than 0.95. All participants were older than 18 years, mostly right-handed (*n* = 45), and had normal or corrected-to-normal vision (with contact lenses only). None of them indicated previous pain-related diseases. Participants received compensation in the form of course credits or monetary compensation (12 € per hour) for their participation. Upon arrival at the laboratory, all participants provided written informed consent in accordance with the Declaration of Helsinki. The study design and all methods were approved by the local ethics committees of the University of Würzburg (GZEK 2023-54). Data were collected between December 2023 and March 2024.

### 2.3 Stimulus materials

**Fig. 1.**
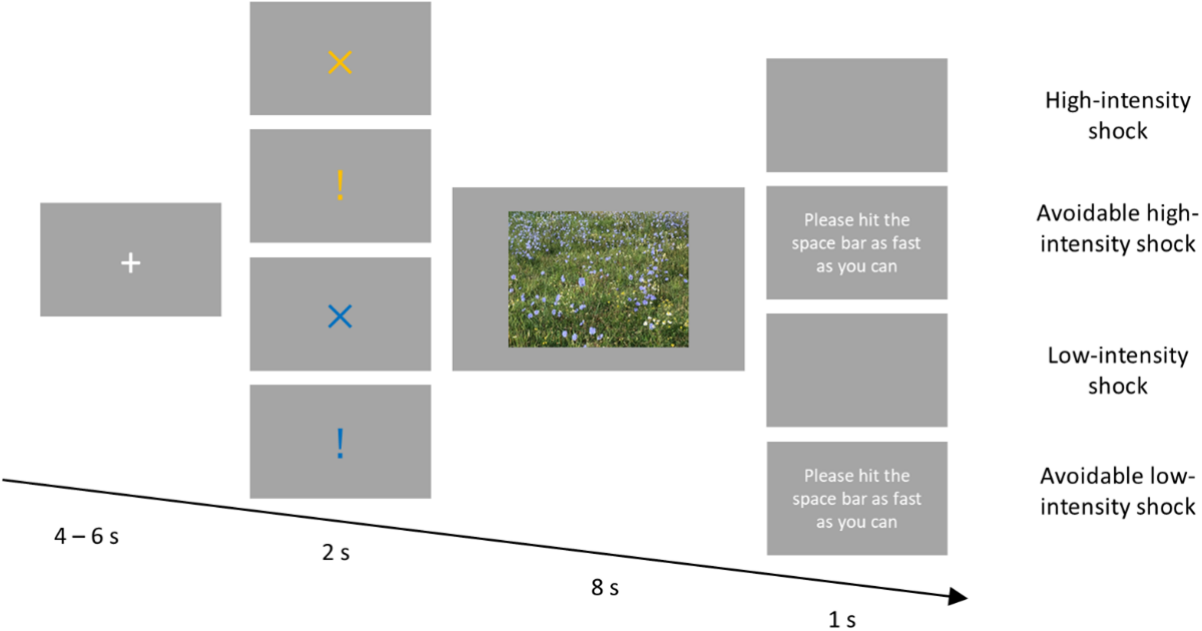
Experimental design and trial structure. Each trial began with a 4–6-second display of a white fixation cross (ITI). Then, a cue was presented for 2 seconds to indicate the response availability (avoidable vs. inevitable) and intensity of the US (high vs. low) for the upcoming condition. Following this, a naturalistic image was shown for 8 seconds. At the end of the image presentation, participants either received a certain US (inevitable conditions) or had the opportunity to avoid the US by quickly pressing the space bar (avoidable threat conditions). Every condition was presented 40 times in random order. Note: The fixation cross and color cues are not drawn to scale.

We used 160 affectively neutral, naturalistic images that were taken from the McGill Calibrated Colour Image Database (*n* = 85), and photographs by the authors (*n* = 75). All images were screened to ensure that they did not contain salient features, such as social cues or a single brightly colored flower standing out against a uniformly green background (see Fig. 1 for an example), as these could potentially limit and guide the participants’ visual exploration behavior. Importantly, each picture was presented only once per participant, and the assignment of pictures to conditions was randomized for each participant. To mitigate attentional biases caused by residual imbalance in visual saliency between the left and right sides of the images, half of the pictures were randomly picked and flipped horizontally for each participant. The stimuli were presented in the center of the screen in front of a gray background on a 17-inch monitor with a resolution of 1280 x 1024 pixels and a refresh rate of 60 Hz. The images spanned visual angles of approximately 14.2° horizontally and 10.0° vertically at a viewing distance of 80 cm. The experimental software *Presentation* (Neurobehavioral Systems, Inc, Albany, CA) was used for stimulus presentation.

The unconditioned stimuli (US) consisted of an aversive electrical pulse train. In order to obtain two different intensities (high intensity—painful; low intensity—unpleasant but not painful), we adjusted the length of the electric pulse train. For the high-intensity condition, we used a 200 ms aversive electrical pulse train (ten 2 ms pulses, separated by 18 ms). For the low-intensity condition, we used one 2 ms pulse only. The US were delivered to the left inner forearm through a surface bar electrode consisting of two stainless-steel disks of 9 mm diameter and 30 mm spacing by a constant current stimulator (Digitimer DS7A, Digitmer Ltd., Welwyn Garden City, UK). Similar to our previous work (Stegmann et al., 2024), US intensities for the high-intensity stimulus were individually adjusted using a staircase procedure. This procedure involved two ascending and descending series of electrical stimuli until a perceived US unpleasantness rating of 6 was achieved on a scale from 0 = “not painful at all” through 4 = “minimally painful” to 10 = “very painful”. After calibration, the mean US intensities were 2.29 ± 1.60 mA (*M* ± *SD*). For the low-intensity stimulation, the same US intensities were used, but the length of the electrical pulse train was reduced as described. This resulted in subjective unpleasantness ratings of 1.92 ± 1.08, indicating a slightly unpleasant experience, well below the pain threshold defined as 4 on the scale.

EEG data were acquired using a 129-electrode Electrical Geodesics high-density EEG System (EGI, Eugene, OR, USA). Gaze position and pupil width were measured using a Tobii Pro Nano eye tracker. Electrodermal activity and ECG were measured using a V-Amp amplifier and the Vision Recorder Software (BrainProducts Inc., Munich, Germany).

### 2.4 Design and procedure

After arrival in the laboratory, participants were informed about the procedure of the study and signed informed consent. Next, the EEG net and sensors for physiological recordings were applied, followed by the individual calibrations of the pain threshold and the eye-tracking system.

The main experiment comprised 160 trials, which were divided into 4 conditions (see Fig. 1; 40 trials per condition). Subjects viewed naturalistic images on a PC screen while their heart rate, skin conductance, eye movements, and electrocortical responses were being recorded. Each trial started with a white fixation cross displayed for 4–6 seconds (ITI). The shape and color of the cue preceding each trial (for 2 seconds) indicated the trial type and predicted the intensity (high vs. low) and response availability (avoidable vs. inevitable) of the electrotactile stimulation following the picture offset. The participants were explicitly instructed about the cue–US contingencies prior to the experiment, including the meaning of each cue with respect to response availability (avoidable vs. inevitable) and shock intensity (high vs. low): A cross signaled a passive (inevitable) trial, where the electrotactile stimulation could not be evaded but had to be endured. An exclamation mark signaled an active (avoidable) trial, where the electrotactile stimulation could be evaded by quickly hitting the space bar upon stimulus offset. The color of the cue (blue vs. orange) predicted the intensity of the shock (high vs. low) with the assignment of colors to intensities counterbalanced across participants.

After the cue, the naturalistic image was presented for 8 seconds. This image period constituted the anticipation phase and was the primary analysis window, as it captured participants’ preparation for a potential response and outcome under threat, while allowing meaningful assessment of oculomotor behavior during viewing of naturalistic scenes. At the offset of the image, participants either received a certain US (inevitable conditions) or had the option to omit the US presentation by quickly pressing the space bar (avoidable conditions) with their right hand. Participants were instructed to respond as quickly as possible on every trial in the avoidable conditions and were informed that shock omission depended on their response time. Trials in which no response occurred within 1 second were excluded from further analyses. This ensured that all analyzed active (avoidable) trials involved action preparation. Similarly, trials in the inevitable condition with erroneous responses were excluded. To ensure that threat avoidance was demanding, the response time threshold was initially set to 250 ms for the first five trials and then dynamically adjusted to the median of the five most recent responses. The median was adjusted separately for the avoidable high- and low-intensity trials. This procedure resulted in an average of 37.3% (*SD* = 7.90%) successful flight responses in the high-intensity and 35.5% (*SD* = 7.69%) in the low-intensity condition. The trial type was randomized with the constraint that no more than three trials of the same condition occurred in succession. The total duration of the experiment, including preparation and filling in the questionnaires, was about 120 minutes. Participants were allowed to take a self-paced break after the first 80 trials.

### 2.5 Measures

#### 2.5.1 Eye tracking

Eye movements and pupil diameter were recorded with a Tobii Pro Nano eye tracker (Tobii Technology, Inc., Tokyo, Japan) at a sampling rate of 60 Hz. Both eyes were recorded, and the mean x- and y-coordinates on the screen, as well as the mean pupil size of both eyes, were stored. Participants sat at a viewing distance of 80 cm with unrestrained head movements. Eye-tracking calibration was done using a nine-point calibration procedure in Presentation before each of the two blocks. Fixations and saccades were detected using an event detection algorithm with a predefined minimum duration of 80 ms and maximum dispersion of 50 pixels (i.e., 0.9° of visual angle) per fixation.

An iterative outlier detection algorithm was applied to ensure that participants fixated on the center of the screen before trial start (Rösler & Gamer, 2019): Fixation locations during the last 300 ms before cue onset were considered as baseline for every trial. The smallest and the largest values were temporarily removed from the distribution of baseline coordinates and marked as outlier if they deviated more than 3 standard deviations from the mean baseline position of the remaining data. Trials with invalid baselines (i.e., substantial deviations from the initial gaze positions of the remaining data) were then excluded, and the procedure was repeated until no more baseline location was marked as outlier. We excluded subjects with more than 50% of missing or outlier baselines (*n* = 6) as well as trials with baseline outliers or missing baseline position data (18.83%) in the remaining data from all further eye-tracking analyses. For the cleaned dataset, the x- and y-coordinates of the baseline were then subtracted from the fixation coordinates during stimulus exploration for an offline drift correction.

Finally, fixations were separated into bins of 1 second each for the 8-second picture viewing period and we calculated three different oculomotor metrics for each bin: the average distance of fixations from the center of the screen in pixels (center bias), the duration of individual fixations, and the number of fixations. This procedure was consistent with our previous research (Merscher et al., 2022; Stegmann et al., 2024).

#### 2.5.2 Pupil width

After extracting pupil diameter from the eye-tracking data, we performed a linear interpolation on segments containing blinks and missing data. Data were kept in its original sampling rate of 60 Hz and original unit (mm). Next, we applied a 2 Hz low-pass filter and conducted a baseline correction by subtracting the mean of the -500 to 0 ms time window, relative to the cue onset, from each ensuing data point. For the statistical analysis, we calculated the average pupil diameter within 20 bins of 0.5 seconds each for every condition.

#### 2.5.3 EDA and ECG

Electrodermal activity (EDA) was recorded using two Ag/AgCl electrodes filled with isotonic (0.5% NaCl) electrolyte medium, placed on the thenar and hypothenar eminences of the left palmar surface. The signal was recorded at a sampling rate of 1,000 Hz with an online notch filter at 50 Hz, using a V-Amp amplifier and the Vision Recorder Software (BrainProducts Inc., Munich, Germany). Offline, the skin conductance data were down sampled to 20 Hz and segmented into time windows from -1,000 to 10,000 ms relative to the cue onset. Changes in skin conductance levels were analyzed by subtracting the mean baseline (-1,000 to 0 ms) from all subsequent data points. These values were then averaged into ten 1-second bins for each condition.

Similarly, the ECG was recorded at a sampling rate of 1,000 Hz using three adhesive Ag/AgCl electrodes placed underneath the right clavicle and the left and right costal margins. Offline, ECG data were down sampled to 100 Hz and filtered using a 2 Hz high-pass filter to remove slow signal drifts. R-peaks were then detected semi-automatically using in-house software (Reutter & Gamer, 2023) and manually edited in case of detection errors. R–R intervals were converted to heart rate (HR) in beats per minute. Changes in heart rate throughout the trials were analyzed relative to a baseline interval from -1,000 to 0 ms prior to trial onset and then averaged into ten 1-second bins for each condition. Mean baseline HR was 74.41 bpm (SD = 9.45), with individual participant values shown in Supplementary Figure S1. Baseline estimates showed good reliability across trials, ICC = .81, 95% CI [.75, .87].

For visualization purposes, the 5 seconds following stimulus offset were also scored for both EDA and heart rate.

#### 2.5.4 EEG

EEG was continuously recorded using a 129-electrode net at a sampling rate of 500 Hz, referenced to Cz. The data were online bandpass filtered with 0.1 and 100 Hz and a 50 Hz notch filter. All impedances were kept below 50 kΩ where possible. Offline processing was conducted in Matlab (The MathWorks Inc., version: 9.2.0, R2017a, Natick, Massachusetts) using the EMEGS (Electro Magnetic Encephalography) software (Peyk et al., 2011). First, epochs of -600 ms to 10,000 ms relative to the cue onset were extracted from the continuous EEG. Data were then filtered with a 40 Hz low-pass filter (23rd-order Butterworth) and a 1 Hz high-pass filter (1st-order Butterworth). Artifact handling was performed using the SCADS procedure (Junghöfer et al., 2000). Trials with artifacts were identified based on statistical parameters, such as absolute value, standard deviation, and maximum differences, at both trial and sensor levels. Contaminated sensors were automatically identified and replaced using statistically weighted, spherical spline interpolated values. Trials with more than 20 contaminated sensors out of 129 were excluded from the analysis. On average, 30.1 ± 11.0% of trials were excluded. Artifact-free trials were then subjected to a time–frequency analysis. Morlet wavelets with a Morlet coefficient of m = 10 were used on artifact-free single trials across a frequency range from 2 to 40 Hz, followed by averaging the single trial time–frequency representations by condition. The resulting native frequency resolution, based on a sampling rate of 500 Hz and 5,300 samples per trial, was 0.0943 Hz. Baseline division was applied using a time segment ranging from -440 to -200 ms. The alpha band was defined as the range between 9.3 and 12.2 Hz for the center frequencies of the Morlet wavelets. Consequently, we obtained a σt = 0.171 and σf = 0.933 for the lowest and σt = 0.131 and σf = 1.216 for the highest frequencies. Changes in alpha power in response to the cues were quantified by averaging the data across sensor Pz and its 7 nearest neighbors (EGI sensors 65, 61, 62, 67, 72, 77, 78, 78) into 20 bins of 500 ms for each condition. Given that previous studies have shown posterior alpha suppression to be most pronounced between 500 and 1,000 ms after the onset of a visual stimulus and following the analysis steps of Stegmann et al. (2024), we focused particularly on these time intervals relative to both the trial onset (500–1,000 ms after cue onset) and the onset of the naturalistic picture (2,500–3,000 ms).

### 2.6 Data analysis

All analyses were conducted in the R software environment (version 4.1.3), using the *ez*-package for ANOVAs (Lawrence & Lawrence, 2016; version 4.4-0). Confidence intervals (95%) for partial eta-squared ($\eta_{p}^{2}$) were calculated with the *MBESS*-package (Kelley, 2020; version 4.9.2).

All dependent variables were analyzed using repeated-measures ANOVAs with the within-subject factors *response availability* (two levels: inevitable vs. avoidable), *intensity* (two levels: high vs. low), and time (0.5 seconds bins for alpha activity and pupil width; 1 second bins for oculomotor measures, heart rate, and SCL). Degrees of freedom were adjusted according to Greenhouse–Geisser to compensate for potential violations of the sphericity assumption (Greenhouse & Geisser, 1959). As pre-registered, we also compared alpha suppression between the conditions in the time windows from 500 to 1,000 ms after visual stimuli onsets, that is, after the onset of the cue as well as after the onset of the naturalistic picture.

For a pair-wise comparison of the conditions throughout the entire trial duration, we applied cluster-based permutation tests (Ehinger, 2016; Maris & Oostenveld, 2007). To do this, we used the binned electrocortical, eye tracking, pupil, heart rate, and skin conductance data and performed pairwise *t*-tests at every time point. Contiguous clusters were identified based on these individual tests. To evaluate the likelihood of such clusters, we shuffled the condition labels within participants and repeated the cluster identification process (1,000 permutations). If the probability of obtaining a cluster as large as ours, or larger, was less than 5% in the absence of any actual difference between conditions, the cluster was considered significant.

To identify predictors of response times in avoidable trials on a trial-by-trial basis, we further computed a generalized linear mixed model (GLMM) including changes in alpha activity, heart rate, pupil dilation, and distance of fixations from the center of the screen as fixed effects and the subject ID as a random intercept (model specification: rt ~ cb + hr + scl + dilation + alpha + (1|subject)). We calculated two separate models for the high- and low-intensity trials, to be able to detect differentiating patterns in the predictors of response time. As in previous studies (Merscher & Gamer, 2024a; Merscher et al., 2022; Rösler & Gamer, 2019; Stegmann et al., 2024), we focused on the second half of the picture presentation, calculating the average responses on a trial-wise basis in an interval between 6,000 and 10,000 ms relative to cue onset. The GLMMs were performed in R, using the *lme4*-package (Bates et al., 2015; version 1.1-31) with a restricted maximum likelihood criterion. We obtained *p*-values for each predictor from t-tests using the Satterthwaite’s approximation of degrees of freedom.

## 3 Results

### 3.1 Oculomotor behavior

**Fig. 2.**
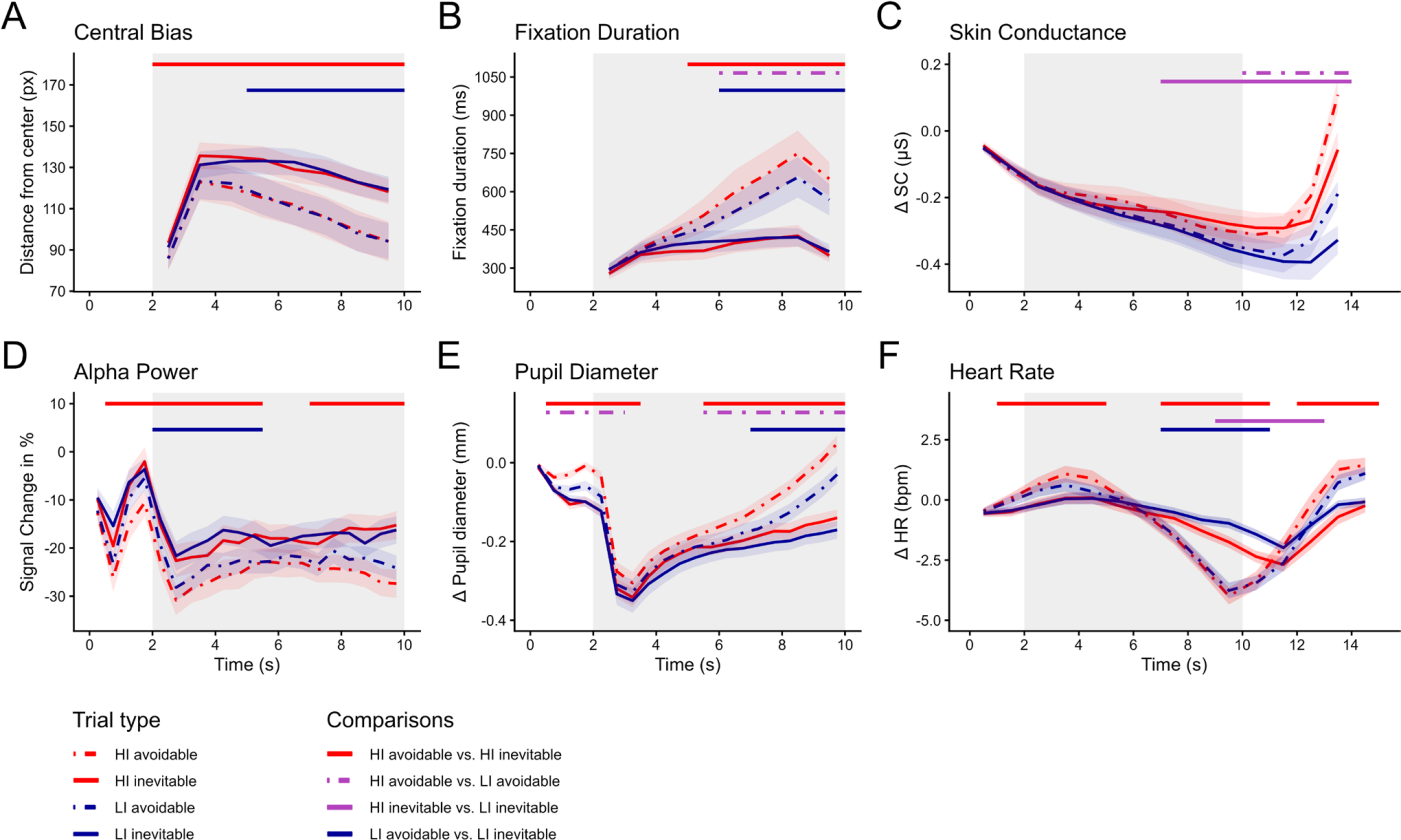
Changes in gaze centralization (A), fixation duration (B), skin conductance level (C), alpha power (D), pupil diameter (E), and heart rate (F) during the anticipation of a high-intensity (red) or low-intensity (blue) electrotactile stimulation that was either avoidable (dot-dashed line) or inevitable (solid line). The cue that informed the participant about the trial type occurred at 0 seconds and was then presented for 2 seconds. Subsequently, the naturalistic pictures were presented from 2 to 10 seconds (light gray shade). The shaded bands denote standard errors of the mean. Colored lines at the top of the figure indicate time points with significant differences between the avoidable and inevitable high-intensity condition (red), avoidable high-intensity and low-intensity condition (purple, dot-dashed line), inevitable high-intensity and low-intensity condition (purple, solid line), and between avoidable and inevitable low-intensity condition (blue), using cluster-based permutation tests. See Supplementary Table S1 for a table containing all clusters detected in the post hoc analysis.

As expected, we found a global center bias in both avoidable conditions. The rmANOVA on the amount of gaze centralization revealed significant main effects for *response availability*, *F*(1, 41) = 9.74, *p* = .003, $\eta_{p}^{2}$ = .19, *CI_9_*_5_ = [.02, .38], and *time bin*, *F*(2.25, 92.34) = 34.63, *p* < .001, $\eta_{p}^{2}$ = .46, *CI_9_*_5_ = [.36, .52], but not for *threat intensity*, *F*(1, 41) = 0.01, *p* = .941, $\eta_{p}^{2}$ < .01, *CI_9_*_5_ = [.00, .04]. Visual exploration decreased, especially toward the end of the anticipation period (interaction between *response availability* and *time, F*(1.95, 79.81) = 4.95, *p* = .010, $\eta_{p}^{2}$ = .11, *CI_9_*_5_ = [.03, .16]); see Fig. 2). No further effect reached statistical significance (*F’*s ≤ 0.71, *p’*s ≥ .566). Post hoc cluster-based permutation tests confirmed significant differences when comparing the avoidable high-intensity condition with inevitable high-intensity trials (2,000–10,000 ms after stimulus onset, *p* < .001) and the avoidable low-intensity condition to inevitable low-intensity trials (5,000–10,000 ms after stimulus onset, *p* = .014). See Supplementary Figure S3 for the normalized fixation density maps reflecting the distribution of fixations for all four conditions.

As a second measure of freezing of gaze, we analyzed temporal fixation characteristics. We observed increased fixation durations in avoidable conditions (main effect *response availability*), *F*(1, 41) = 9.97, *p* = .003, $\eta_{p}^{2}$ = .20, *CI_9_*_5_ = [.03, .39], as well as across the trial time (main effect *time bin*), *F*(1.45, 59.38) = 30.15, *p* < .001, $\eta_{p}^{2}$ = .42, *CI_9_*_5_ = [.33, .48]. This increase of fixation durations over time was further influenced by both *response availability* (interaction between *response availability* and *time, F*(1.23, 50.62) = 15.08, *p* < .001, $\eta_{p}^{2}$ = .27, *CI_9_*_5_ = [.17, .33]), and *intensity* (interaction between *intensity* and *time, F*(3.17, 129.80) = 4.15, *p* = .007, $\eta_{p}^{2}$ = .09, *CI_9_*_5_ = [.02, .14]). The factor *intensity* alone yielded a marginally significant effect, *F*(1, 41) = 2.98, *p* = .092, $\eta_{p}^{2}$ = .07, *CI_9_*_5_ = [.00, .24]. In addition, there was also a significant interaction between *response availability* and *intensity, F*(1, 41) = 8.92, *p* = .005, $\eta_{p}^{2}$ = .18, *CI_9_*_5_ = [.02, .37]. Specifically, when comparing the two inevitable conditions, threat intensity did not significantly affect fixation durations (*M*_high vs. low_ = −13.8 ± 67.8 ms). However, in the avoidable conditions, the high-intensity shock led to a further increase in durations compared with the low-intensity condition (*M*_high vs. low_ = 49.9 ± 119 ms). This interaction was further modulated by the factor time as evident by a significant three-way interaction, *F*(3.44, 141.03) = 2.81, *p* = .035, $\eta_{p}^{2}$ = .06, *CI_9_*_5_ = [.01, .10], see Figure 2. Looking at the post hoc comparisons, a consistent increase in fixation durations can be observed when comparing avoidable with passive inevitable trials in the low-intensity conditions (6,000–10,000 ms after cue onset, *p* = .011) as well as in the high-intensity conditions (5,000–10,000 ms after cue onset, *p* = .005). Further, high- and low-intensity trials differed significantly in avoidable trials (6,000–10,000 ms after cue onset, *p* = .003). A similar pattern was observed for the number of fixations, indicating consistent effects across these two measures of gaze behavior (see Supplementary Material and Supplementary Figure S2).

### 3.2 Autonomic responses

#### 3.2.1 Pupil diameter

The ANOVA for changes in pupil diameter revealed significant differences between avoidable and inevitable trials (main effect *response availability*, *F*(1, 47) = 15.99, *p* < .001, $\eta_{p}^{2}$ = .25, *CI_9_*_5_ = [.07, .43]), high- and low-intensity trials (main effect *intensity*, *F*(1, 47) = 7.99, *p* = .007, $\eta_{p}^{2}$ = .15, *CI_9_*_5_ = [.01, .32]), and time (main effect *time bin*, *F*(2.40, 112.66) = 75.65, *p* < .001, $\eta_{p}^{2}$ = .62, *CI_9_*_5_ = [.57, .64]). In addition, we found that these temporal dynamics of the pupil diameter are further modulated by both *response availability* (interaction between *response availability* and *time, F*(2.56, 120.54) = 26.67, *p* < .001, $\eta_{p}^{2}$ = .36, *CI_9_*_5_ = [.30, .40]), and *intensity* (interaction between *intensity* and *time*, *F*(2.72, 127.87) = 3.52, *p* = .020, $\eta_{p}^{2}$ = .07, *CI_9_*_5_ = [.02, .09]), and there was also a three-way interaction between these factors *F*(3.90, 183.48) = 2.67, *p* = .035, $\eta_{p}^{2}$ = .05, *CI_9_*_5_ = [.01, .07]. The interaction of *intensity* and *response availability* was the only effect that did not reach statistical significance (*F*(1, 47) = 1.53, *p* = .223). Post hoc cluster-based permutation tests revealed almost consistent increases in pupil diameter, when comparing the avoidable high-intensity condition with inevitable high-intensity trials (500–3,500 ms after cue onset, *p* = .040; 5,500–10,000 ms after cue onset, *p* = .022), but also with the avoidable low-intensity trials (500–3,000 ms after cue onset, *p* = .030; 5,500–10,000 ms after cue onset, *p* = .007), starting already during the cue period (see Fig. 2).

#### 3.2.2 Heart rate

**Fig. 3.**
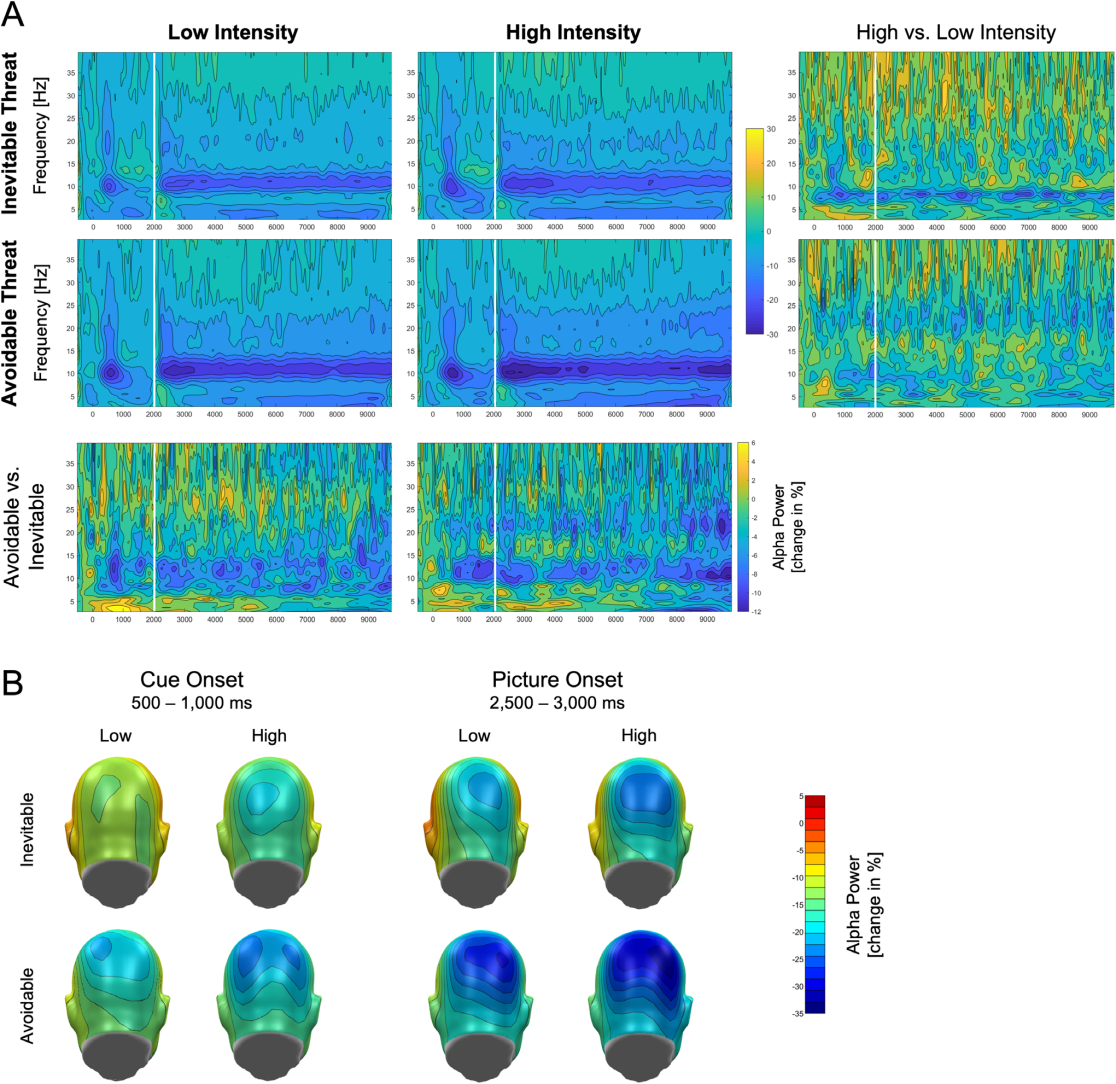
Changes in parieto-occipital alpha power. Time–frequency plots (A) for inevitable threat vs. avoidable threat and low-intensity vs. high-intensity trials as well as the differences between the respective plots. The respective topographies (B) at two different time windows: 500–1,000 ms relative to cue onset; 500–1,000 ms relative to picture onset (i.e., 2,500–3,000 ms relative to cue onset). Power values are percent changes relative to the baseline. The cue appeared at 0 ms. The naturalistic pictures were presented from 2,000 to 10,000 ms. The white line marks the onset of the naturalistic picture.

Analysis of the heart rate responses revealed a significant main effect of *time bin*, *F*(2.62, 123.12) = 62.09, *p* < .001, $\eta_{p}^{2}$ = .57, *CI_9_*_5_ = [.50, .61]. The overall biphasic response pattern across the trial time was modulated by both *response availability* (interaction between *response availability* and *time, F*(2.83, 133.16) = 45.86, *p* < .001, $\eta_{p}^{2}$ = .49, *CI_9_*_5_ = [.42, .54]) and *intensity* (interaction between *intensity* and *time*, *F*(3.46, 162.62) = 5.15, *p* = .001, $\eta_{p}^{2}$ = .10, *CI_9_*_5_ = [.04, .14]). Specifically, in both avoidable conditions, the heart rate showed an initial increase, peaking 3 seconds after cue onset, followed by a deceleration toward the end of the trial (see Fig. 3). Additionally, there was a more pronounced fear bradycardia in the inevitable high-intensity condition compared with the inevitable low-intensity condition toward the end of the picture presentation and beyond the offset of the picture (9,000–13,000 ms after cue onset, *p* = .019). No other effects in the repeated-measures ANOVA reached statistical significance (*F’*s ≤ 1.34, *p’*s ≥ .253).

#### 3.2.3 Skin conductance

The repeated-measures ANOVA revealed that skin conductance levels were higher in the two high-intensity threat conditions than in the low-intensity conditions (Fig. 2; main effect *intensity F*(1, 47) = 4.67, *p* = .036, $\eta_{p}^{2}$ = .09, *CI_9_*_5_ = [.00, .26]) and interaction *intensity* x *time*, *F*(1.98, 92.85) = 8.35, *p* < .001, $\eta_{p}^{2}$ = .15, *CI_9_*_5_ = [.08, .20]). Post hoc tests revealed that skin conductance levels during the inevitable high-intensity condition differed significantly from those in the inevitable low-intensity condition toward the end of the anticipation period (7,000–10,000 ms after cue onset, *p* = .038). No other cluster reached statistical significance. In addition, there was a significant main effect of *time bin*, *F*(1.17, 55.19) = 39.46, *p* < .001, $\eta_{p}^{2}$ = .46, *CI_9_*_5_ = [.38, .50], indicating a general decrease of skin conductance across the trial.

### 3.3 Electrocortical activity

**Fig. 4.**
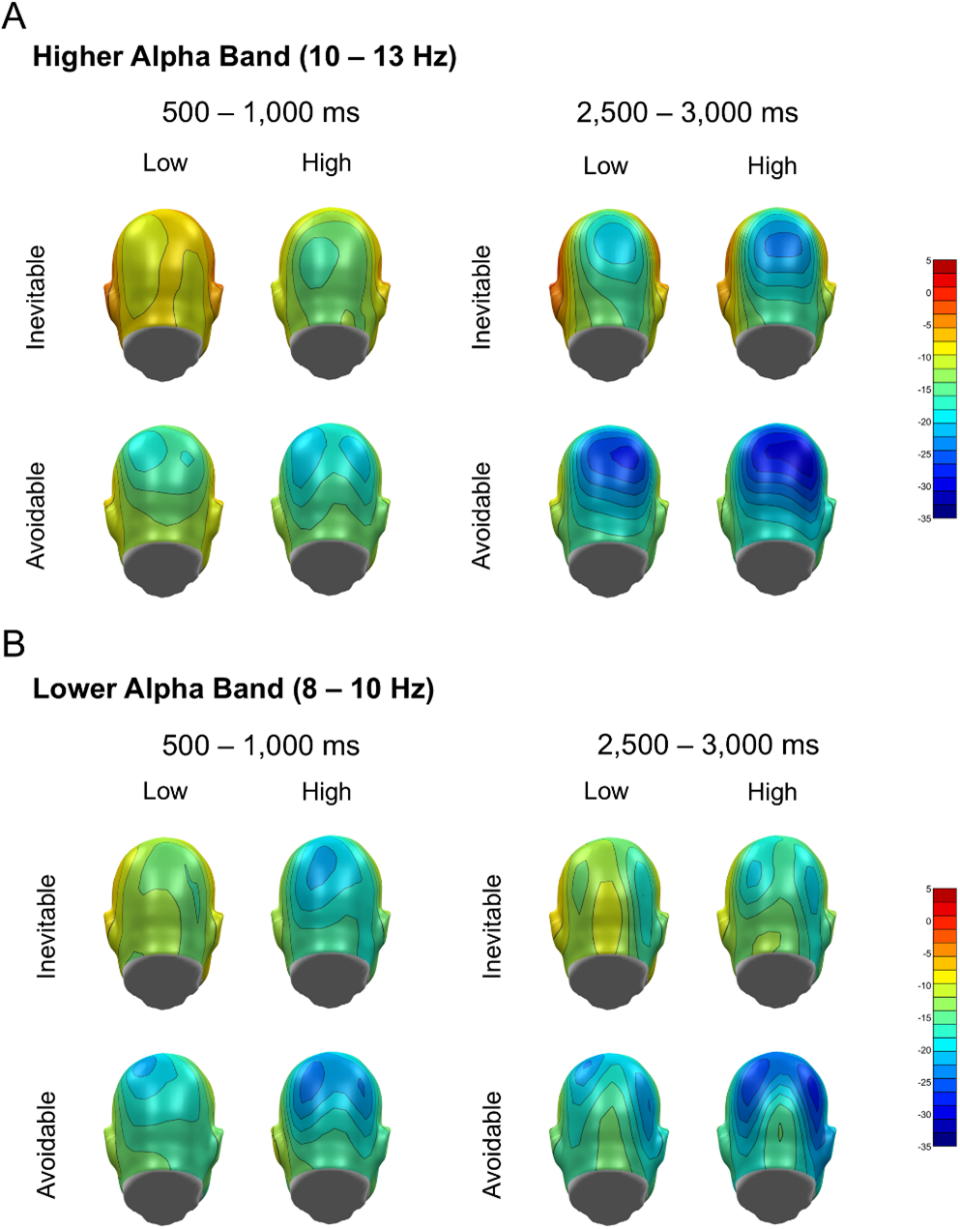
The topographies for inevitable threat vs. avoidable threat and low-intensity vs. high-intensity trials at two different time windows: 500–1,000 ms relative to cue onset; 500–1,000 ms relative to picture onset (i.e., 2,500–3,000 ms relative to cue onset), separately for the higher alpha band (A) and the lower alpha band (B). Power values are percent changes relative to the baseline.

For changes in parieto-occipital alpha suppression throughout the trial, the repeated-measures ANOVA revealed significant main effects of *response availability*, *F*(1, 47) = 18.02, *p* < .001, $\eta_{p}^{2}$ = .28, *CI_9_*_5_ = [.08, .45], and *time bin*, *F*(3.76, 176.69) = 20.37, *p* < .001, $\eta_{p}^{2}$ = .30, *CI_9_*_5_ = [.24, .33]. The corresponding time–frequency plots are shown in Figure 4. When repeating the ANOVA for our predefined time points, we found that the cue onset induced a stronger alpha suppression between 500 and 1,000 ms in the avoidable as compared with inevitable trials, *F*(1, 47) = 17.33, *p* < .001, $\eta_{p}^{2}$ = .27, *CI_9_*_5_ = [.08, .45], as well as in the high-intensity compared with low-intensity trials, *F*(1, 47) = 5.26, *p* = .026, $\eta_{p}^{2}$ = .10, *CI_9_*_5_ = [.00, .27]. Similarly, the onset of the naturalistic picture prompted stronger alpha suppression (2,500–3,000 ms) in the avoidable than in the inevitable conditions, *F*(1, 47) = 19.32, *p* < .001, $\eta_{p}^{2}$ = .29, *CI_9_*_5_ = [.09, .45]. However, threat intensity did not significantly affect alpha suppression during this interval (see Fig. 2). Consequently, in the cluster-based permutation test we observed significant differences when comparing the avoidable high-intensity condition with inevitable high-intensity trials (500–5,500 ms, *p* = .003; 7,000–10,000 ms after stimulus onset, *p* = .012) and the avoidable low-intensity condition with inevitable low-intensity trials (2,000–5,500 ms after stimulus onset, *p* = .006).

### 3.4 Avoidance behavior

In avoidable high-intensity trials, the participants’ mean response time was 286.74 ms (*SD* = 52.96 ms), whereas they were significantly slower in the low-intensity condition with a mean response time of 308.44 ms (*SD* = 55.36 ms), *t*(47) = −5.53, *p* < .001, *d* = −0.80 [−1.12; −0.47]. The generalized linear mixed model for the high-intensity trials revealed that faster response times were associated with an increased suppression of alpha activity, a more pronounced heart rate deceleration, and an increase in pupil diameter. In contrast, in the low-intensity trials, faster response times were predicted by an increased skin conductance level and a more pronounced heart rate deceleration (see Table 1). An increased center bias, that is, decreased distances of fixations from the center of the screen, did not predict faster response times beyond the other significant predictors in the model in any of the two trial types.

**Table 1.** Parameters of the generalized linear mixed models predicting response times in avoidable threat trials.

	High-intensity trials				Low-intensity trials			
Variable	β	*SE*	*t*	*p*	β	*SE*	*t*	*p*
Alpha	10.87	3.45	3.15	**.002**	8.15	4.52	1.80	.072
CB	3.08	3.00	1.03	.304	6.36	4.02	1.58	.114
Pupil	-7.16	3.27	-2.19	**.029**	-3.96	3.91	-1.01	.311
SCL	1.24	2.86	0.433	.665	-11.68	3.86	-3.02	**.003**
HR	9.70	2.93	3.31	**< .001**	13.36	3.84	3.48	**< .001**

Note. CB: center bias; HR: heart rate; SCL: skin conductance level. *p*-Values for each predictor were obtained based on Satterthwaite’s approximation of degrees of freedom.

Bold *p*-values indicate statistically significant effects (*p* < .05).

### 3.5 Exploratory analysis of electrocortical data

A visual inspection of the time–frequency plots in Figure 3 suggested that the differences between conditions are not uniformly distributed across the entire alpha range. Instead, two distinct sub-bands within the alpha range show variation between conditions: higher alpha power (above 10 Hz) appears particularly relevant for distinguishing between avoidable and inevitable conditions, whereas a narrower band below 10 Hz appears to differentiate rather between high- and low-intensity conditions. Based on these observations, we conducted an exploratory analysis by separating the alpha range into upper (10–13 Hz) and lower alpha (8–10 Hz) bands.

#### 3.5.1 Alpha power suppression in the higher alpha band (10–13 Hz)

For the higher alpha band, parieto-occipital alpha suppression varied significantly with *response availability*, *F*(1, 47) = 31.72, *p* < .001, $\eta_{p}^{2}$ = .40, *CI_9_*_5_ = [.19, .56], and *time bin*, *F*(3.49, 163.91) = 35.49, *p* < .001, $\eta_{p}^{2}$ = .43, *CI_9_*_5_ = [.37, .46]. No further effect reached statistical significance. During the predefined intervals between 500 and 1,000 ms after the onset of a visual stimulus, we found a stronger alpha suppression in the avoidable than in the inevitable trials following cue onset (500–1,000 ms: *F*(1, 47) = 28.79, *p* < .001, $\eta_{p}^{2}$ = .38, *CI_9_*_5_ = [.16, .54]) and picture onset (2,500–3,000 ms: *F*(1, 47) = 30.88, *p* < .001, $\eta_{p}^{2}$ = .40, *CI_9_*_5_ = [.18, .55]). However, threat intensity did not significantly influence suppression in this band during either interval. The topographic distribution of higher alpha suppression closely resembled that of the whole alpha range (see Fig. 4).

In line with the strong effect of response availability, high alpha band power reduction predicted response times on a trial-wise basis, when added to the generalized linear mixed model. However, similar to when adding the whole alpha range, this was only the case for high-intensity trials (β = 9.26, *t*(707.79) = 2.41, *p* = .016), but not for low-intensity trials (β = −1.17, *t*(713.94) = −0.22, *p* = .827).

#### 3.5.2 Alpha power suppression in the lower alpha band (8–10 Hz)

For the lower alpha band, suppression varied significantly with *response availability*, *F*(1, 47) = 7.30, *p* < .001, $\eta_{p}^{2}$ = .13, *CI_9_*_5_ = [.01, .31], *threat intensity*, *F*(1, 47) = 9.10, *p* = .004, $\eta_{p}^{2}$ = .16, *CI_9_*_5_ = [.02, .34], and *time bin*, *F*(4.55, 214.05) = 8.19, *p* < .001, $\eta_{p}^{2}$ = .15, *CI_9_*_5_ = [.09, .17]. Following cue onset (500–1,000 ms), alpha suppression was stronger in avoidable versus inevitable trials, *F*(1, 47) = 5.52, *p* = .023, $\eta_{p}^{2}$ = .11, *CI_9_*_5_ = [.00, .28], and in high- versus low-intensity trials, *F*(1, 47) = 12.63, *p* < .001, $\eta_{p}^{2}$ = .21, *CI_9_*_5_ = [.04, .39]. Similar patterns were found following picture onset (2,500–3,000 ms): suppression was greater in avoidable versus inevitable conditions, *F*(1, 47) = 20.37, *p* < .001, $\eta_{p}^{2}$ = .30, *CI_9_*_5_ = [.10, .47], and in high- than in the low-intensity trials, *F*(1, 47) = 8.36, *p* = .006, $\eta_{p}^{2}$ = .15, *CI_9_*_5_ = [.01, .33]. Interestingly, and in contrast to the results from the whole alpha band and the high alpha band, effects were overall strongest for the factor *intensity.* When looking at the respective topographies (see Fig. 4), it is thus not surprising that power reduction in the low alpha band varies not only with response availability but also strongly with the intensity of the threat. Topographically, reductions in lower alpha power differed notably from those in the higher alpha band. While higher alpha showed a more central reduction, lower alpha suppression was more lateralized across parietal regions, particularly during picture onset.

These findings align with the stronger role of threat intensity in modulating lower alpha power, which may explain its limited association with response times. When added to the generalized linear mixed model, we found no significant associations for lower alpha reductions and response times, neither for high-intensity trials (β = 3.09, *t*(702.37) = 0.87, *p* = .383), nor for low-intensity trials (β = −4.00, *t*(702.11) = −0.93, *p* = .352).

## 4 Discussion

The aim of the current study was to investigate electrocortical, autonomic, and oculomotor responses during defensive states in humans. Specifically, we sought to discern response patterns during states of attentive immobility that are specifically tied to perceived threat levels, primarily linked to processes of action preparation, or influenced by both factors. To achieve this, we adapted a previously used experimental paradigm (Rösler & Gamer, 2019; Stegmann et al., 2024) by varying shock intensities to manipulate threat perception. Participants observed naturalistic images with cues preceding each trial indicating whether to expect an inevitable high-intensity painful shock, inevitable low-intensity shock, avoidable high-intensity shock, or avoidable low-intensity shock after the picture offset. We hypothesized that attentive immobility would vary based on both threat intensity and response availability in the situation, as these factors together influence motivational relevance.

Our findings effectively replicate those of prior studies using the original paradigm (e.g., Rösler & Gamer, 2019; Stegmann et al., 2024). Specifically, when individuals had the opportunity to escape an aversive stimulus in a threatening situation, markers of attentive immobility—such as alpha suppression, focused gaze behavior, pupil dilation, and heart rate deceleration—were more pronounced. Importantly, several of these markers, specifically heart rate deceleration, pupil dilation, and alpha power reduction, further predicted individual response times on a trial-wise, within-subject basis. This suggests that the currently observed response pattern does not indicate a passive behavioral shutdown but rather reflects an adaptive state preparing the individual for a potential escape. Additionally, fixation durations, pupil dilation, and heart rate bradycardia across the trial were further modulated by the threat intensity or an interaction thereof. Only skin conductance was modulated by threat intensity alone.

Although an effect of threat intensity was observed for some measures, this effect was largely overshadowed by the effect of action preparation—the ability to act and avoid the threat. Consistent with previous evidence (Rösler & Gamer, 2019), these results suggest that physiological responding is shaped more strongly by the opportunity to act than by threat intensity alone. Additionally, the discovery that freezing-like patterns can predict faster actions (e.g., Rösler & Gamer, 2019; Stegmann et al., 2024) highlights the role of these processes in enhancing selective attention toward critical information, a function previously suggested by Lojowska et al. (2015). In our study, we identified several components of attentive immobility that were predictive of successful flight reactions. Consistent with earlier research, heart rate bradycardia emerged as a significant predictor of response times across both high- and low-intensity flight trials, suggesting that heart rate deceleration is a multifaceted response, not limited to defensive behavior (e.g., Jennings et al., 1998; Merscher et al., 2022; Reyes del Paso et al., 2015). In their systematic review, Skora et al. (2022) even propose that threat-related heart rate deceleration is best viewed as a subset of attentive anticipation during action preparation, challenging the distinction between threat-related and action-anticipatory heart rate deceleration altogether. In contrast, the fact that alpha suppression and pupil dilation predicted response times only in the more aversive trials suggests that these physiological markers play a critical role in action preparation in the face of threat.

Taken together, our findings reveal a distinct difference in the strength of the attentive immobility response, particularly between avoidable and inevitable threats, underscoring the motivational significance of threat stimuli in shaping these effects. These results align with the concept that attentive immobility represents an active state, which facilitates action preparation in the face of avoidable threats (Roelofs, 2017) and supports findings from Gladwin et al. (2016), who observed that markers of attentive immobility, including reduced body sway and heart rate deceleration, were evident only during anticipation when participants had the means to defend themselves from a threat, rather than when they were unarmed and helpless. Collectively, these findings substantiate the notion that threat perception and action preparation are inherently linked and serve as a core function of the human defense system (Stegmann et al., 2024). Both threat intensity and response availability appear to enhance motivational relevance and optimize coping responses to threat, as evidenced by faster reaction times under conditions of higher threat intensity. This connection between threat processing and action readiness is essential for survival: as soon as a potential threat is detected, the body engages systems that prime an individual for rapid movement, be it to ‘freeze for action’, flee, or confront the threat. Autonomic changes, such as heart rate deceleration and pupil dilation, work alongside behavioral and neural mechanisms to sharpen focus, prepare the muscles, and optimize the body’s readiness for immediate action. Only in hopeless situations, tonic immobility—accompanied by a lack of preparation for action—is used as a last resort for survival (Fanselow, 1994; Mobbs et al., 2020; Volchan et al., 2017). Interestingly, even in situations without an escape option, Fullana et al. (2016) found consistent activation in the supplementary motor area, suggesting that motor responses are primed but then inhibited when action is impossible. Thus, defensive responding might be intrinsically tied to motor preparatory processes, reflecting a deeply ingrained survival mechanism. Viewing threat responding as a process primarily driven by motivational relevance likely explains why the effects observed in the current threat anticipation paradigm closely parallel findings from studies on perception and action in non-threatening contexts: Heart rate deceleration has been associated with enhanced processing of the external environment in different studies where subjects anticipated action (Lacey & Lacey, 2008; Skora et al., 2022). A reduced heart rate is thought to facilitate external information processing by reducing the impact of systolic baroreceptor activation (Skora et al., 2022). For instance, a stronger heart rate deceleration but also a reduced alpha power during action preparation has been associated with superior performance in aiming sports, such as golf, archery, and darts, and was linked to general performance accuracy (Cooke, 2013; Henderson et al., 2024). Similarly, a more focused (“frozen”) gaze has been found to predict performance across studies on the “Quiet Eye effect” in aiming sports (Lebeau et al., 2016). Our results, therefore, complement other research on autonomic, electrocortical, and behavioral data in highly motivated anticipatory states not necessarily tied to a threatening context.

Exploratory analysis revealed that alpha modulation was not uniform across the frequency range but instead showed distinct patterns in two sub-bands. Alpha power in the upper frequency band (10–13 Hz) was modulated exclusively by action preparation, with suppression topographically centered over occipitoparietal regions. In contrast, lower alpha power (8–10 Hz) was sensitive to both action readiness and the perceived intensity, with a more lateralized distribution of the suppression across parietal regions. While this distinction was not anticipated, similar results have been reported in other studies, which also identified functional dissociations between lower and upper alpha bands (Klimesch et al., 1998; Krause et al., 2000; Verstraeten & Cluydts, 2002). Specifically, lower alpha frequencies are thought to reflect general task demands, such as overall alertness, or arousal, whereas upper alpha is more closely linked to specific task requirements, such as semantic memory or task-focused attention (Klimesch et al., 1998; Verstraeten & Cluydts, 2002). This interpretation aligns with our findings across the sub-bands and might explain their differential ability to predict response times. Other studies, however, report consistent results across the entire alpha and lower beta range, with reductions in power from 8 to 20 Hz in response to emotional scenes and highly arousing pictures (Ferrari et al., 2020; Schubring & Schupp, 2021), but also in classical fear conditioning studies in response to the CS+ (Bacigalupo & Luck, 2022), peaking around 10 Hz. In their review on alpha band oscillations, Codispoti et al. (2023) summarized that strong responses across these frequencies generally occur when the motivational significance of stimuli is high. Thus, overall alpha suppression appears to be modulated by the motivational relevance, which in our study comprises both, threat intensity and response availability. Given the post hoc and exploratory nature of these analyses, the present findings should be considered preliminary. While the pattern observed here tentatively suggests that lower alpha may be sensitive to both threat intensity and action readiness, whereas upper alpha may be more closely related to response availability and action preparation, independent preregistered replications are required to further substantiate this interpretation.

It seems important to speculate whether the observed pattern, with a pronounced effect of action preparation, may partly reflect limitations in our study design. The use of 160 trials, often involving over 80 electrotactile stimulations, could have led to habituation effects, reducing the impact of threat intensity. Despite recalibrating shock intensity during the break between blocks, overall intensity ratings declined across the experimental blocks. Conversely, even though low-intensity stimulations were rated as non-painful, most participants still found them aversive, which may have heightened their motivation to avoid these stimuli even in low-intensity trials. The observed differences in response times between high- and low-intensity trials confirm that we successfully manipulated avoidance motivation. However, they also highlight the challenge of fully disentangling the effects of threat perception and action preparation, as preparation seems to increase with threat intensity. Consequently, our results regarding threat intensity are not solely attributable to the perceived threat but might also be explained by increased action preparatory processes or higher motivation and effort, showing again that these processes are inherently linked. Another limitation concerns the relatively short intertrial intervals (4–6 seconds), which were optimized for EEG signal quality and trial count. As a consequence, skin conductance responses may not have fully returned to baseline between trials, resulting in a gradual downward drift. Although this timing is suboptimal for tonic SCL recovery, baseline correction and the clear condition differences suggest that the measure still provides meaningful information.

Overall, our findings indicate that attentive immobility to approaching threats involves a multimodal response across neural, autonomic, and behavioral levels, all of which enhance perceptual processing and facilitate action preparation. The interplay between these components during attentive immobility suggests a need to move away from the notion of separate systems governing defensive and motor responses under threat. Instead, we propose an integrative framework, where responses to potential threats are driven primarily by their motivational relevance, which increases with factors such as perceived imminence, harm potential, and, crucially, the possibility of avoidance. These findings offer new perspectives on defensive behavior, emphasizing the integrated nature of fear responses and action preparation for future research in humans and other species.

## Supplementary Material

Supplementary Material

## Data Availability

This study’s design, all hypotheses, and analyses were preregistered in December 2023 at https://osf.io/2kp4s. Aggregated datasets and the code for all statistical analyses are available on the Open Science Framework at https://osf.io/f9hjx/. Data were collected from December 2023 to March 2024.
