# Supplementary material for: Acting upon threat: Electrocortical, autonomic, and oculomotor responses in anticipation of avoidable and inevitable threats
Source: Imaging Neurosci (Camb). 2026 Jul 29;4:IMAG.a.1318. doi: 10.1162/IMAG.a.1318 (PMC13449923; doi:10.1162/IMAG.a.1318)
Supplement: Supplementary file 1 [file IMAG.a.1318_supp.pdf]

Supplementary Materials

Heart rate baseline values

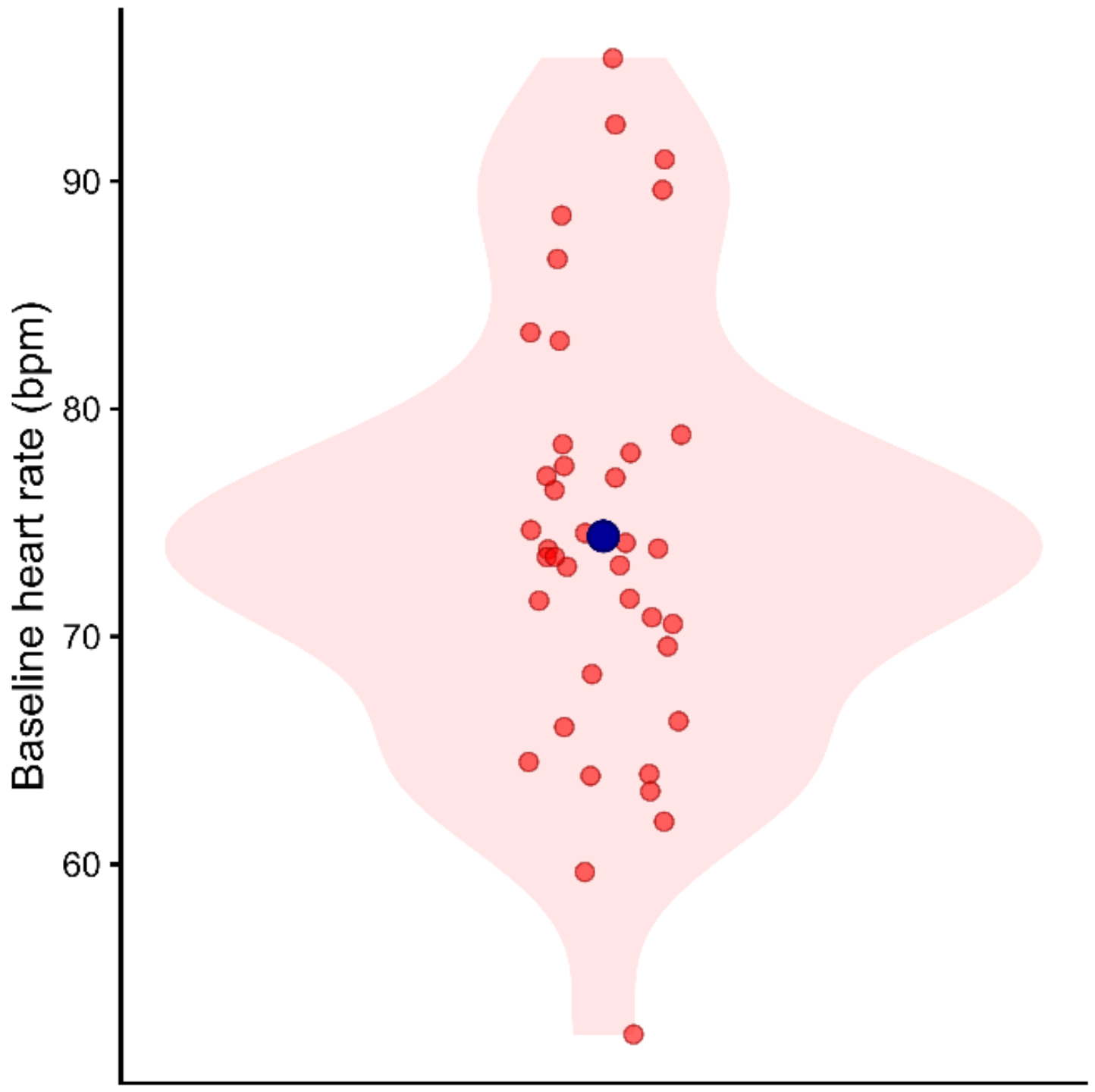

Figure S1. Participant-level baseline heart rates. The violin plot illustrates the distribution of participant-specific base-line heart rate estimates, calculated as the mean baseline heart rate across trials for each participant. Individual jittered points represent participants, and the blue marker denotes the grand mean baseline heart rate across participants.

Number of Fixations

In addition to fixation duration, we also analyzed the number of fixations. The overall pattern of the two metrics was very similar. We observed decreased fixation numbers in active conditions (main effect *response availability*), $F(1, 41) = 6.00$, $p = .019$, $\eta_p^2 = .13$, $CI_{95} = [.00, .32]$, as well as across the trial time (main effect *time bin*), $F(2.72, 111.34) = 37.06$, $p < .001$, $\eta_p^2 = .47$, $CI_{95} = [.38, .53]$. The decrease in number of fixations over the course of the trial time further differed between avoidable and inevitable conditions (interaction of *response availability* and *time bin)*, $F(2.93, 120.22) = 30.14$, $p < .001$, $\eta_p^2 = .42$, $CI_{95} = [.33, .48]$.

Additionally, we observed a marginally significant main effect of *intensity*, $F(1, 41) = 3.87$, $p = .056$, $\eta_p^2 = .09$, $CI_{95} = [.00, .27]$ and a significant interaction between *response availability* and *intensity,* $F(1, 41) = 7.66$, $p = .008$, $\eta_p^2 = .16$, $CI_{95} = [.01, .35]$. Specifically, in the two inevitable conditions, threat intensity did not significantly affect the number of fixations ($M = 0.010 \pm 0.206$ fixations/sec), whereas in the avoidable conditions, the high-intensity shock led to a further decrease ($M = -0.109 \pm 0.221$ fixations/sec). No further interaction reached significance (Fs ≤ 1.79, ps $\geq .116$).

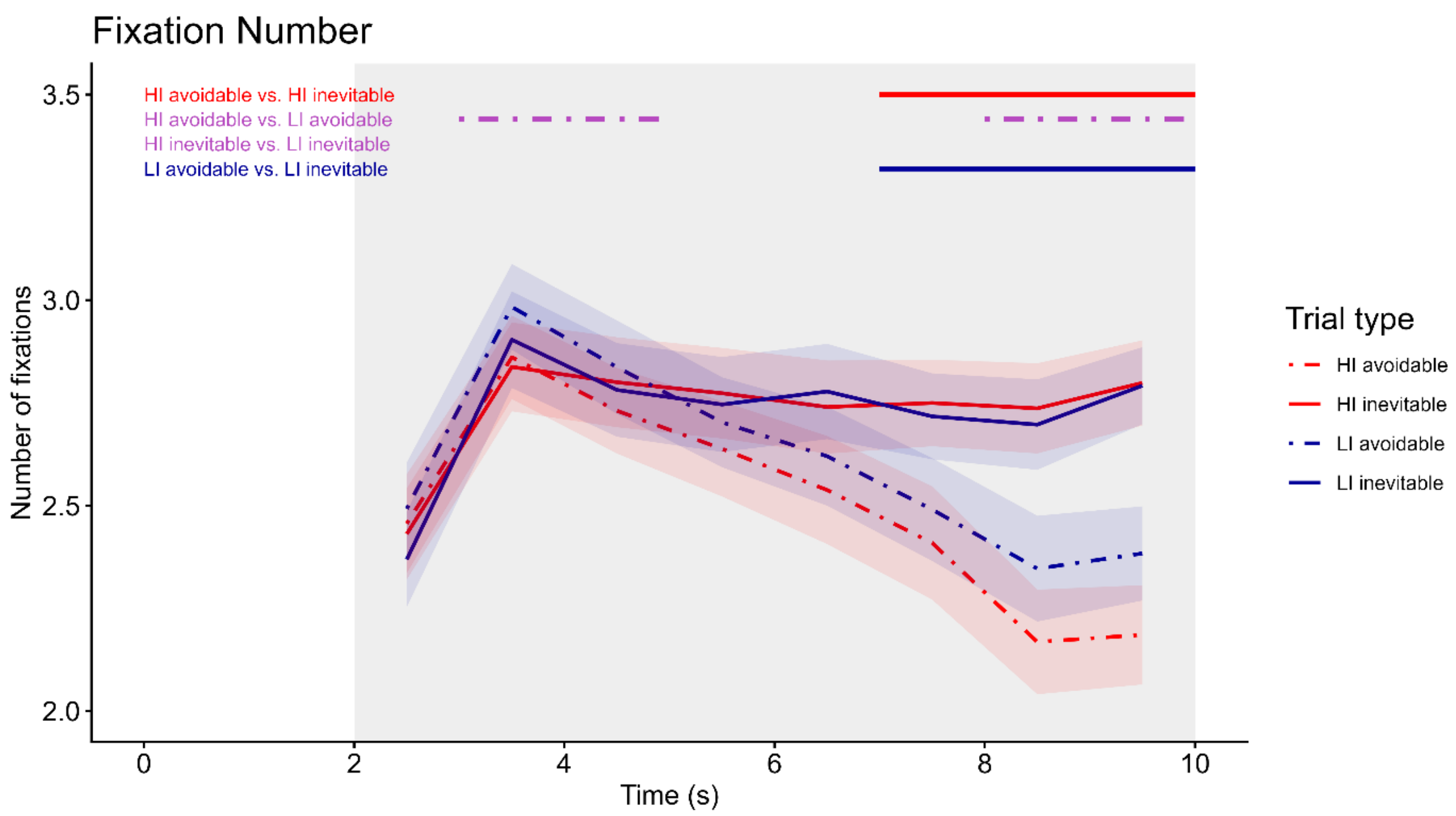

Figure S2. Changes in the number of fixations during the anticipation of a high-intensity (red) or low-intensity (blue) electrotactile stimulation that was either avoidable (dot-dashed line) or inevitable solid line). The shaded bands denote standard errors of the mean. Colored lines at the top of the figure indicate time points with significant differences between the avoidable and inevitable high-intensity condition (red), avoidable high-intensity and low-intensity condition (purple, dot-dashed line), inevitable high-intensity and low-intensity condition (purple, solid line), and between avoidable an inevitable low-intensity condition (blue), using cluster-based permutation tests. See Table S1 for a table containing all clusters detected in the post-hoc analysis.

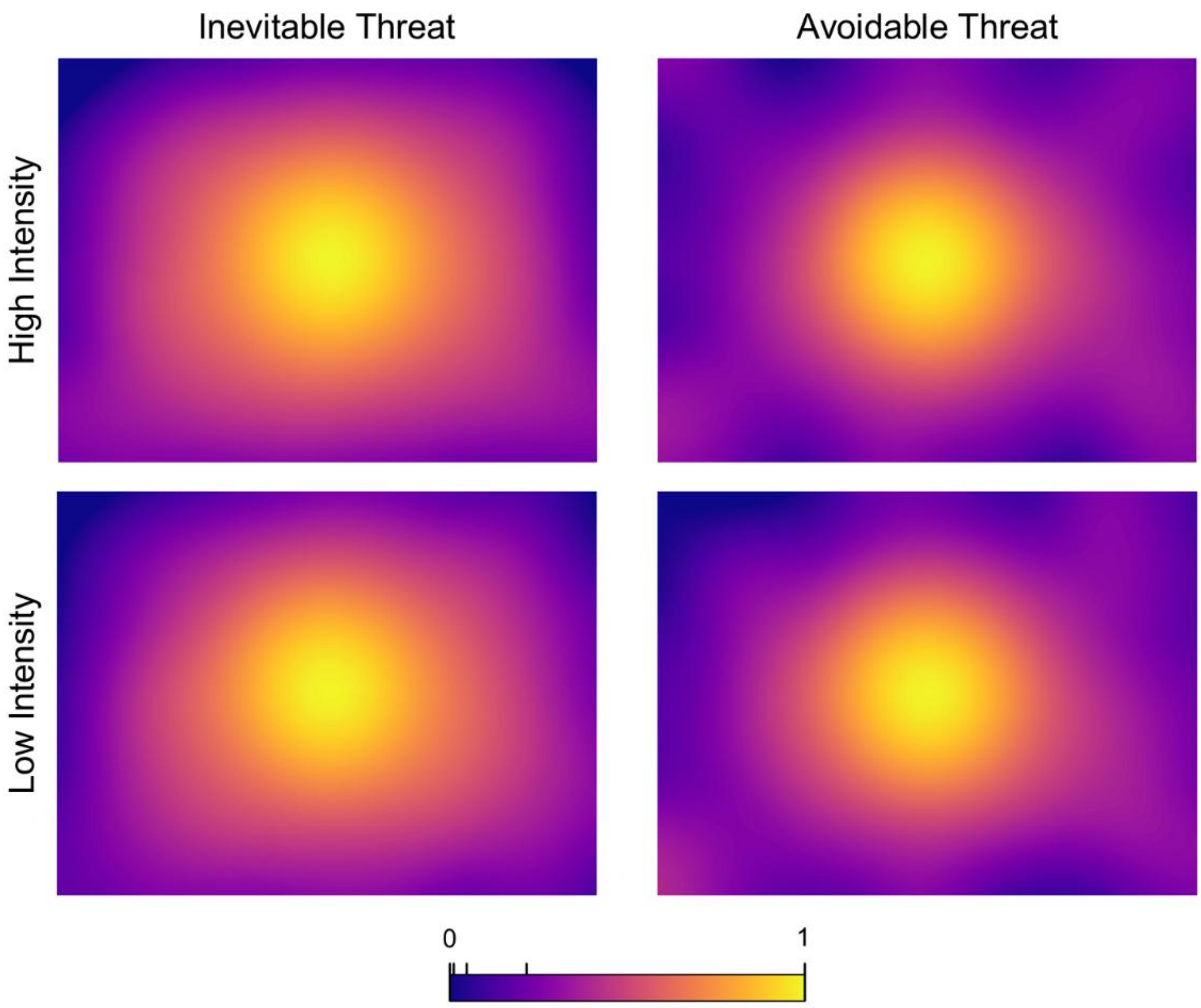

Figure S3. Normalized fixation density maps as a function of response availability and intensity. Maps reflect the distribution of fixations on the images during the anticipation phase on a logarithmic scale.

Post-hoc bin-wise comparisons between conditions

Table S1 - Results of the cluster-based permutation tests

DV	Comparison	Cluster nr	start	end	length	Critical length	*p* value
SCL	Inevitable: high vs. low	1	8	14	7	4	**.013**
	Avoidable: high vs. low	1	11	14	4	4	**.045**
	High: avoidable vs. inevitable	1	14	14	1	4	.105
	Low: avoidable vs. inevitable	1	13	14	2	3	.069
HR	Inevitable: high vs. low	1	10	13	4	3	.019
	Avoidable: high vs. low	1	12	13	2	3	.072
	High: avoidable vs. inevitable	1	2	5	4	3	**.017**
	High: avoidable vs. inevitable	2	8	11	4	3	**.017**
	High: avoidable vs. inevitable	3	13	15	3	3	**.045**
	Low: avoidable vs. inevitable	1	3	4	2	3	.090
	Low: avoidable vs. inevitable	2	8	11	4	3	**.019**
	Low: avoidable vs. inevitable	3	14	15	2	3	.090
Pupil	Avoidable: high vs. low	1	1	3	5	4	**.030**
	Avoidable: high vs. low	2	6	10	9	4	**.007**
	High: avoidable vs. inevitable	1	1	3.5	6	5	**.040**
	High: avoidable vs. inevitable	2	6	10	9	5	**.022**
	Low: avoidable vs. inevitable	1	1.5	2.5	3	4	.062
	Low: avoidable vs. inevitable	2	7.5	10	6	4	**.024**
CB	High: avoidable vs. inevitable	1	3	10	8	4	**<.001**
	Low: avoidable vs. inevitable	1	6	10	5	2	**.014**
FNum	Avoidable: high vs. low	1	4	5	2	2	**.011**
	Avoidable: high vs. low	2	9	10	2	2	**.011**
	High: avoidable vs. inevitable	1	8	10	3	2	**.027**

	Low: avoidable vs. inevitable	1	8	10	3	2	**.019**
FDur	Inevitable: high vs. low	1	3	3	1	2	.066
	Inevitable: high vs. low	2	6	6	1	2	.066
	Avoidable: high vs. low	1	7	10	4	2	**.003**
	High: avoidable vs. inevitable	1	6	10	5	2	**.005**
	Low: avoidable vs. inevitable	1	7	10	4	2	**.011**
Alpha	Avoidable: high vs. low	1	1.5	1.5	1	3	.124
	High: avoidable vs. inevitable	1	1	5.5	10	3	**.003**
	High: avoidable vs. inevitable	2	7.5	10	6	3	**.012**
	Low: avoidable vs. inevitable	1	1	1	1	3	.127
	Low: avoidable vs. inevitable	2	2.5	5.5	7	3	**.006**
	Low: avoidable vs. inevitable	3	7.5	7.5	1	3	.127
	Low: avoidable vs. inevitable	4	9.5	10	2	3	.061

Note. CB: center bias; HR: heart rate; SCL: skin conductance level; FNum: number of fixations; FDur: duration of fixations. Clusters could range from 0 to 10 seconds with the first two seconds representing the cue phase and the remaining eight seconds comprising the anticipation phase. Data were analyzed in 1 s bins for oculomotor measures, heart rate, and SCL, and in 0.5 s bins for alpha activity and pupil width.
